# Understanding Hazardous Materials Transportation Accidents Based on Higher-Order Network Theory

**DOI:** 10.3390/ijerph192013337

**Published:** 2022-10-16

**Authors:** Cuiping Ren, Bianbian Chen, Fengjie Xie, Xuan Zhao, Jiaqian Zhang, Xueyan Zhou

**Affiliations:** 1 School of Modern Posts, Xi’an University of Posts and Telecommunications, Xi’an 710061, China; 2 Key Laboratory of Transportation Industry of Automotive Transportation Safety Enhancement Technology, Chang’an University, Xi’an 710064, China

**Keywords:** higher-order network, hazardous materials transportation accident, accident causation analysis, topological properties

## Abstract

In hazardous materials transportation systems, accident causation analysis is important to transportation safety. Complex network theory can be effectively used to understand the causal factors of and their relationships within accidents. In this paper, a higher-order network method is proposed to establish a hazardous materials transportation accident causation network (HMTACN), which considers the sequences and dependences of causal factors. The HMTACN is composed of 125 first- and 118 higher-order nodes that represent causes, and 545 directed edges that denote complex relationships among causes. By analyzing topological properties, the results show that the HMTACN has the characteristics of small-world networks and displays the properties of scale-free networks. Additionally, critical causal factors and key relationships of the HMTACN are discovered. Moreover, unsafe tank or valve states are important causal factors; and leakage, roll-over, collision, and fire are most likely to trigger chain reactions. Important higher-order nodes are discovered, which can represent key relationships in the HMTACN. For example, unsafe distance and improper operation usually lead to collision and roll-over. These results of higher-order nodes cannot be found by the traditional Markov network model. This study provides a practical way to extract and construct an accident causation network from numerous accident investigation reports. It also provides insights into safety management of hazardous materials transportation.

## 1. Introduction

Hazardous materials are essential materials for economic development and life and are mostly transported by road. In China, road transport volume is about 1.2 billion tons per year, which accounts for 69% of all transport modes. However, hazardous materials during transportation are a mobile dangerous source, especially in road transportation. In China, more than 80% of hazardous material transportation accidents occur during road transportation [1], in which the average growth rate from 2013 to 2018 was 1.9% [2].

To reduce the accidents, it is essential to identify their key causes and causative paths. Accidents are usually caused by unsafe factors including humans, vehicles, hazardous materials, environment, and management, and their causations are complex due to the interaction of factors. For example, on 13 June 2020, there was a major accidental explosion of liquefied petroleum gas transportation, causing 20 deaths and 175 injuries with a loss of more than 94.77 million RMB in Zhejiang, China (6/13 Major Explosion Accident). The cause of this accident was the driver’s failure to slow down in time, resulting in the tank hitting a guardrail and rapidly releasing LPG. Simultaneously, the sparks from passing motor vehicles ignited the LPG, resulting in a steam cloud explosion [3]. This indicates that the causes of hazardous materials transportation accidents can be characterized by time sequences, dependences, and serious consequences.

There are many methods for describing the accident causation during hazardous material transportation, e.g., sequential, epidemiological, and system models. The sequence model regards an accident as the result of a series of discrete and unexpected events [4,5,6,7,8,9]. However, the sequence model fails to consider the coupling effect among factors. The epidemiological models, e.g., energy transfer theory, Swiss cheese model, and Human Factors Analysis and Classification System (HFACS), all suggest that an accident usually results from both potential and explicit defects, and analyze accident causes from individual and organizational aspects. Li et al. introduced an HFACS method based on the Bayesian network (BN-HFACs) and found that hazardous material environment (1.63) and mechanical equipment (0.49) in the level of preconditions of unsafe behavior had the same direction failure effect as operation error [10]. This model does not consider the interactions and evolutions of accident causes. The system model incorporates complex social technology system theory to analyze the behavior of the whole system and the fundamental causes. This model holds the view that an accident is caused by wrong decisions and actions from all layers of the system, which emphasizes the progressive structure of accident factors at different layers [11]. The system model mainly focuses on qualitative analysis, which is not conducive to revealing the quantitative relationships of a complex accident causation system.

Accidents involving hazardous materials road transportation are the result of coupled multifactor effects. However, those aforementioned methods have not yet revealed the interactions among the factors. Hazardous materials road transportation can be regarded as a complex system that consists of five subsystems, namely hazardous materials, personnel, vehicles, environment, and management. Each subsystem contains many factors. Once a factor experiences a problem, this unsafe factor will affect its related factors, then put the system in a dangerous state. Complex network theory has an advantage in revealing the topological characteristics of complex systems. This method has been widely used to analyze accident causation for railways [12,13,14,15] and subways [16,17]. The common practice to construct an accident causation network is based on a pairwise relationship model [18]. Accidents and unsafe factors are abstracted as nodes, and the causal relationships among them are represented as edges [14]. However, this direct conversion is based on the assumption of the Markov property (first-order dependency [19]), which losses important information about data dependences from raw data [18].

In recent years, on the basis of complex network theory, higher-order networks have been proposed that can obtain momentous information on data dependency [20]. The higher-order network [21,22,23,24,25,26], which considers time-series, can record where nodes come from and how they proceed. Scholars have proved that a high-order network is significantly better than the conventional first-order network in identifying key nodes from the aspects of model structure [27]. Additionally, higher-order dependencies have been shown to either change node ranking [28,29] or alter community structures [18,22,30,31,32,33]. Xu et al. emphasized that it is important to consider the non-Markovian process when modeling sequential data and proposed a higher-order network representation and algorithm [18,34]. Lotito et al. defined higher-order motifs as small connected subgraphs in which vertices may be linked by interactions of any order and proposed an efficient algorithm to extract complete higher-order motif profiles from empirical data [35]. Aktas et al. proposed two new Laplacians that allowed redefining classical graph centrality measures for higher-order interactions [36]. Vasilyeva et al. studied higher-order scientific collaboration networks where a single link can connect more than two individuals [37]. Causal factors are sequential and dependent, so non-Markovian higher-order models are appropriate for constructing networks of hazardous materials transportation accidents. Based on this network, the dependence among accident factors can be analyzed, which is important for identifying key causal factors and higher-order interactions. Higher-order networks have been used in the Chinese high-speed railway system [38] and will be applied to more research fields.

The rest of this paper is organized as follows: In Section 2, a higher-order network model of hazardous materials transportation accidents (HMTAs) occurring during road transport is introduced. Section 3 shows the results of key causal factors and paths by analyzing topological properties of the HMTACN. Finally, the paper is concluded in Section 4.

## 2. Methodology

### 2.1. Data and Data Analysis

Many accidents are under-reported and or reporting is incomplete; for example, a technical report by the National Traffic Safety Administration [39] estimated that 25% of minor injury accidents and half of no-injury crashes are unreported [40], and only the accidents with detailed information on causes are collected and analyzed. In this study, accident data were mainly collected from the Petrochemical Accident Analysis and Data Interpretation Platform (PAADIP). In PAADIP, if accident reports lack causal information, then the detailed information is further gathered from the Internet. Finally, 792 accidents, all of which happened in hazmat road transportation in China from 2017 to 2021, were analyzed.

To construct accident causation network and make a comprehensive analysis of accident causes, causal factors should first be identified. Usually, the causal factors can be classified into 6 general causes in a systemic way: “Human (H)”, “Vehicles (V)”, “Environment I”, “Hazardous materials (HM)”, “Management (M)”, and “Accident type (A)”. As this analysis is conducted on hazardous materials that have an important impact on the occurrence of an accident, hazardous materials (HMs) are set as one of causal factors. Each classification of causal factors has various specific causes. For example, human causal factors mainly refer to unsafe behaviors during the transportation of hazardous materials, such as fatigued driving, distracted driving, fast driving, improper braking, etc. The specific causes are presented in Table 1.

Accidents may be triggered by simultaneous actions with more than one factor. Thus, the combined interaction of causal factors is considered as multiple factors that have not been analyzed before. Multiple factors can be described by combining the codes of single factors. For example, the multifactor of E03 and H03 can be marked as E3H3.

Based on the aforementioned causal factors shown in Table 1, accident chains were identified through accident reports. These accident chains captured the full complexity of multiple relationships among different types of HMTAs. For example, the chain of 6·13 Major Explosion Accident is driver’s failure→hit the guardrail→LPG released and sparks from passing motor vehicles→steam cloud explosion. All causal chains were extracted from accidents.

### 2.2. Construction of HMTACN

A network is composed of nodes and edges. Nodes represent components, and edges represent the complex correlations among nodes [41]. Considering the dependencies and the time sequences of causal factors in HMTAs, an accident causation network for hazardous materials road transportation was constructed based on a non-Markovian higher-order model. In this network, nodes represent specific causes, and edges represent interrelationships among causes. Edges are extracted from accident chains. With the BuildHON algorithm [34], the process of this network contains two parts, which are the extraction of path dependency and process of network construction.

#### 2.2.1. Extraction of Path Dependency

**Figure 1:**
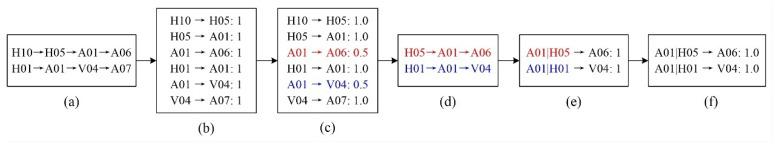
Path dependency extraction of HMTA chains. (**a**) Two accident chains. (**b**) The frequency of all subpaths. (**c**) The transfer probability from node *i* to node *j*. (**d**) The subpaths of A01→A06 and A01→V04 were extended to H05→A01→A06 and H05→A01→V04. (**e**) The frequencies of second-order subpaths. (**f**) The transfer probability of second-order path dependency.

To illustrate the extraction of path dependency, two HMTAs chains were exemplified. Accident chain 1 is represented by H10→H05→A01→A06, and accident chain 2 is represented by H01→A01→V04→A07, as shown in Figure 1a.

In HMTAs chains, a first-order subpath represents the sequence of any two adjacent nodes, e.g., H10→H05, H05→A01, etc. Based on the statistical data of HMTAs, all first-order subpaths were abstracted. Then, the frequency of all subpaths was calculated. As shown in Figure 1b, six first-order subpaths were abstracted with the frequency of 1.

Next, the transfer probability P(i→j) from node i to node j is calculated with Formula (1). And W(i→j) means the frequency of i to j.

(1) $$P\left(i\to j\right)=\frac{W\left(i\to j\right)}{\sum_{h}W\left(i\to h\right)}$$

In Figure 1c, based on Formula (1), the transfer probability was calculated, which was P (H10→H05)=P (H05→A01)=P (H01→A01)=P (V04→A07)=1/1=1.0. This transfer probability indicated that these transfers were definite. Node A01 may point to nodes A06 and V04 with a frequency of 1. So, P(A01→A06)=P(A01→V04)=1/(1+1)=0.5 per Formula (1). The transfer probabilities of A01→A06 and A01→V04 were 0.5, which indicated that these transitions were indefinite, and higher-order path dependencies increased.

Then, the subpaths of A01→A06 and A01→V04 were extended to H05→A01→A06 and H01→A01→V04, as shown in Figure 1d. The frequencies of second-order subpaths A01|H05→A06 and A01|H01→V04 were calculated as 1, as shown in Figure 1e. The transfer probability of second-order subpath P(i|g→j) was calculated by Formula (2). W(i|g→j) means the frequency of subpath i→j coming from g.

(2) $$P\left(i|g\to j\right)=\frac{W(i|g\to j)}{\sum_{h}W(i|g\to h)}$$

The transfer probabilities of A01|H05→A06 and A01|H01→V04 were both 1.0, as shown in Figure 1f. Obviously, this is greater than the transfer probabilities of A01→A06 and A01→V04 (i.e., 0.5). Such an increase in transition probabilities from 0.5 to 1.0 is significant by comparing the Kullback–Leibler divergence $D_{KL}$ with a dynamic threshold δ [39]. As a result, the second-order path dependencies of A01|H05→A06 and A01|H01→V04 were established.

As the transfer probabilities of second-order path dependency was 1.0 without any higher dependencies, the growth of dependency stopped. By abstracting path dependency from HMTAs chains, six first-order nodes (H10, H05, A06, H01, V04, A07) and two second-order nodes (A01|H05, A01|H01) were obtained. H10→H05, H05→A01, H01→A01, and V04→A07 were named first-order path dependencies, and A01|H05 and A01|H01 were named second-order path dependencies. For brief expression, dependencies that were second- and higher-order were named higher-order path dependencies, and the corresponding node was named a higher-order node.

#### 2.2.2. Process of Network Construction

**Figure 2:**
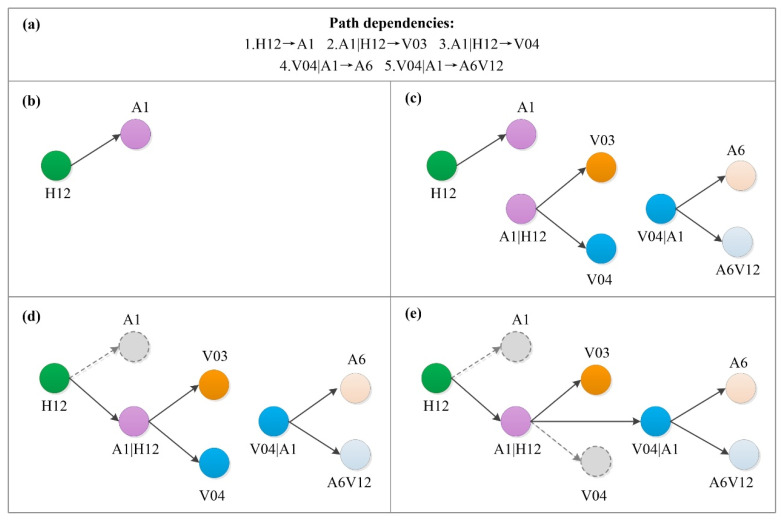
Network construction of HMTACN. (**a**) Path dependencies. (**b**) The first-order path dependency (H12→A1) is converted into two first-order nodes (H12, A1) and their edges. (**c**) The higher-order path dependencies are converted into higher-order nodes and their edges. (**d**) Reconnect the higher-order node from H12 to A1|H12. (**e**) Reconnect the higher-order node from A1|H12 to V04|A1.

Based on the above extraction of path dependency, the HMTACN’s construction was performed by using some path dependencies, as shown in Figure 2a.

Firstly, the first-order nodes with their corresponding edges were formed by converting first-order path dependency. For example, H12→A1 was converted into H12 and A1 with their edges, as shown in Figure 2b.

Then, the higher-order nodes with their outgoing edges were formed by converting higher-order path dependency. For example, A1|H12→V03 and A1|H12→V04 were converted into a higher-order node A1|H12 and two outgoing edges. Meanwhile, V04|A1→A6 and V04|A1→A6V12 were also transferred in the same way, as shown in Figure 2c.

Finally, we reconnected the higher-order nodes. For example, H12 pointed to A1, so the edge from H12 to A1 was reconnected to A1|H12, as shown in Figure 2d. Additionally, the edge from A1|H12 to V04 in Figure 2e was reconnected to V04|A1.

## 3. Results and Discussion

### 3.1. Overall Description of the HMTACN

**Figure 3:**
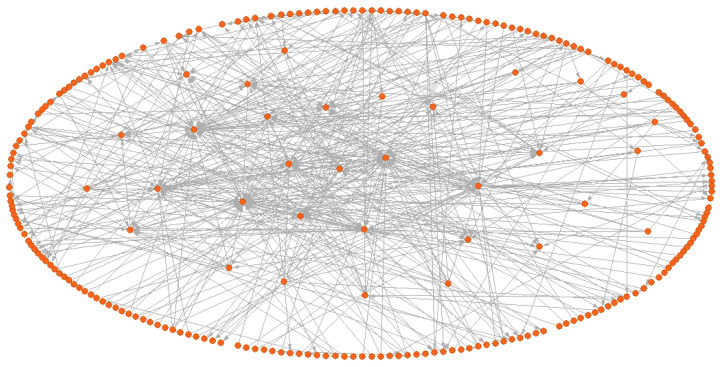
The overall topology of HMTACN.

**Figure 4:**
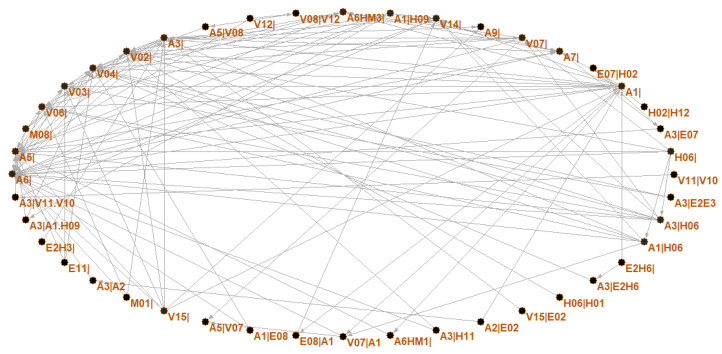
Partial topology of HMTACN.

Based on the aforementioned method in Section 2.2, the HMTACN was constructed. The HMTACN consisted of 243 nodes and 545 directed edges. These 243 nodes contain first-order nodes and higher-order nodes. Figure 3 shows the overall topology of the HMTACN by using Pajek. Pajek is software for analyzing and visualizing network structures. There were too many nodes and edges to clarify the detailed information on labels and the corresponding relationships in Figure 3. Therefore, a partial topology of the HMTACN is represented in Figure 4.

In Figure 4, we can see 21 first-order nodes, such as A1|, A3|, V12|, V14|, A6HM1|, and E2H3|, and 24 higher-order nodes, such as H02|H12, V08|V12, A3|E2E3, and A3|V11.V10. Different higher-order nodes may represent how different path dependencies point to the same node, such as A3|E2E3 and A3|V11.V10. A3|E2E3 reflects a continuous interaction from E2E3 to A3. A3|V11.V10 illustrates A3 is directed from V11, and V11 is directed from V10. To reveal the characteristics of HMTACN, topology properties were analyzed as described below.

### 3.2. Topological Properties

#### 3.2.1. Degree Distribution

**Figure 5:**
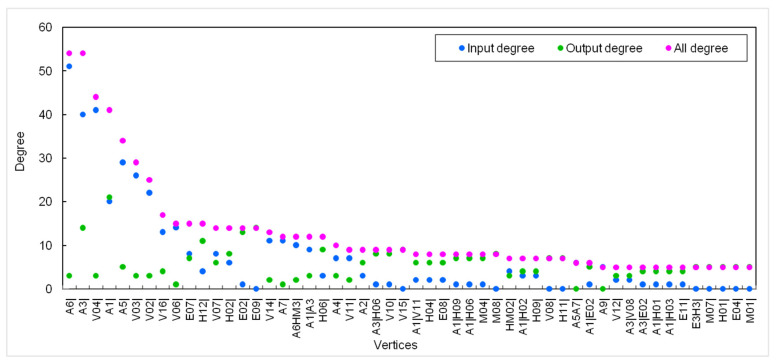
The nodes whose values of all 0 degree are more than 5.

The degree of a node in a network is the number of edges connected to the node. It is the simplest and most important property in a network. The average degree value in the HMTACN was 4.49, indicating that each causal factor connected to four or five factors on average. For directed networks, degree can be classified as input, output, and total degrees. A node with a greater input degree is more affected by other nodes; a node with a greater output degree is more likely to affect others. As there were 243 nodes in the HMTACN, it was necessary to focus on the higher-degree nodes. Therefore, Figure 5 only illustrates the nodes whose degrees were more than five.

As shown in Figure 5, A6|(Leakage), A3|(Roll-over), V04|(Tank damaged), A1|(Collision), A5|(Fire), V03|(Valve damaged), and V02|(Valve loose) have a relatively high all-degree with values of 54, 54, 44, 41, 34, 29, and 25, respectively. This means that the above node factors play significant roles in the road transportation system of hazardous materials, because these nodes can directly affect or be affected by others. A6| (Leakage) has the greatest all degree (54) and input degree (51), while the value of output degree is only 3. This indicates 54 causes can directly lead to the leakage of hazardous materials. Nodes with greater input degrees, such as A3|(Roll-over), V04|(Tank damaged), A5|(Fire), V03|(Valve damaged), V02|(Valve loose), V16|(Packaging issues), and V06|(Pipe rupture), can be easily influenced. A1|(Collision), A3|(Roll-over), E09|(Rain or snow weather), and E02|(Slippery road) have a high output degree with values of 21, 14, 14, and 13, respectively. E09|(Rain or snow weather) and E02|(Slippery road) have a greater output degree than input degree. This indicates weather and roads usually interact with other factors. What is interesting is that human causal factors are not notably high. This is different from another study [42], which argued that human factors (26.74%) are the main cause of hazmat transportation accidents by simply using statistical analysis without considering the interactions among causal factors.

**Figure 6:**
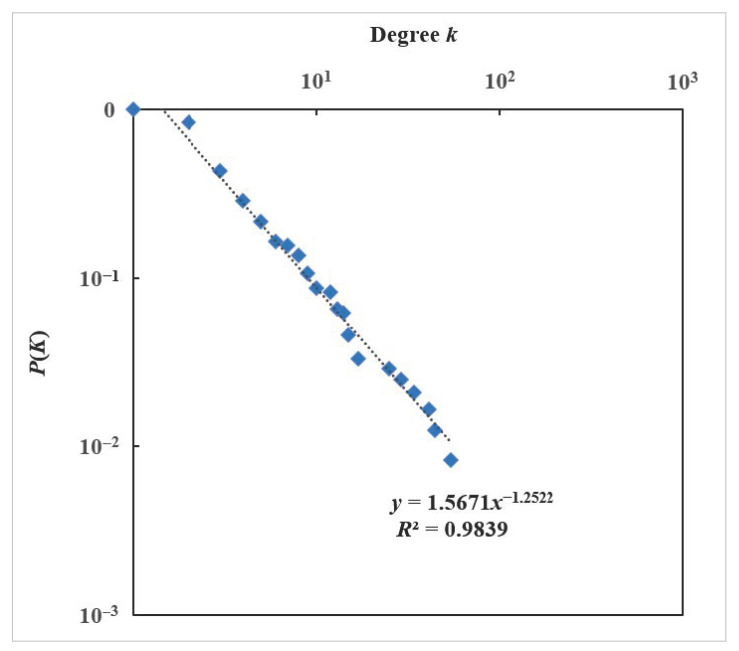
Cumulative degree distribution of HMTACN.

The cumulative degree distribution of the HMTACN follows an exponential function, $P\left(K\right)\sim 1.5671\times k^{-1.2522}\left(R^{2}=0.9838\right)$, as shown in Figure 6. This means that the HMTACN is in line with a scale-free network power-law distribution. A few important causal nodes account for the high degree value. For example, the top 21.4% causal nodes account for the majority (62.2%) of the whole causal relations, and most causal nodes only have 1~3 causal relations. So, if nodes with high degree are attacked, the HMTACN will become fragile and change into a set of independent subnetworks.

#### 3.2.2. Average Path Length and Diameter

Average path length is defined as the average number of steps along the shortest paths for all possible pairs of nodes in a network. A network with a short average path length has high transmission efficiency. In the HMTACN, the average path length is 4.97, which indicates that connecting one accident cause to another only requires five steps. However, in a random network with the same scale and average degree, the value of average path length is 6.26. This indicates that HMTAs have a lower average path length and are more likely to happen than common accidents. The diameter of a network is defined as the longest path among all of the shortest paths in a network. In the HMTACN, the diameter is 12; in a random network, the diameter is 16. This indicates that in the HMTACN, the accident factors are more closely related than in a random network.

#### 3.2.3. Clustering Coefficient

The clustering coefficient is an important method usually employed to describe which nodes in a network prefer to gather together. The clustering coefficient of a network indicates the average clustering coefficient of all the nodes. The value of the clustering coefficient in the HMTACN is 0.0526, which is more than that of a random network (0.0105) with a similar scale and average degree. Compared with the random network, the HMTACN has a shorter average path length and larger clustering coefficient. This indicates that the HMTACN is a small-world network [43], which is consistent with the findings of common accident research [15,16]. This implies that most accident causes can be affected by others through a small number of steps. It is a challenge to prevent the spreading of unsafe factors in the HMTACN.

#### 3.2.4. Betweenness Centrality

**Figure 7:**
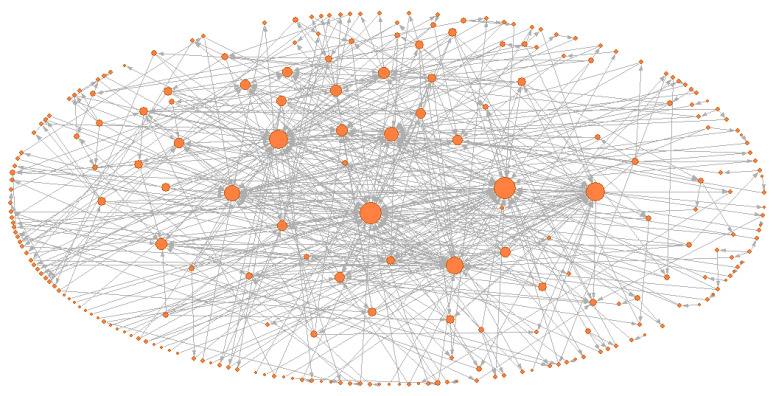
Betweenness centrality of HMTACN.

The betweenness centrality of nodes refers to the proportion of geodesic curves passing through this node among all other nodes. It reflects the importance of nodes as intermediaries. The higher the betweenness centrality of nodes, the greater the role of information transmission in the network. A node with high betweenness centrality has important influences on the transfer of items through the network. Figure 7 shows the betweenness centrality of the HMTACN.

**Figure 8:**
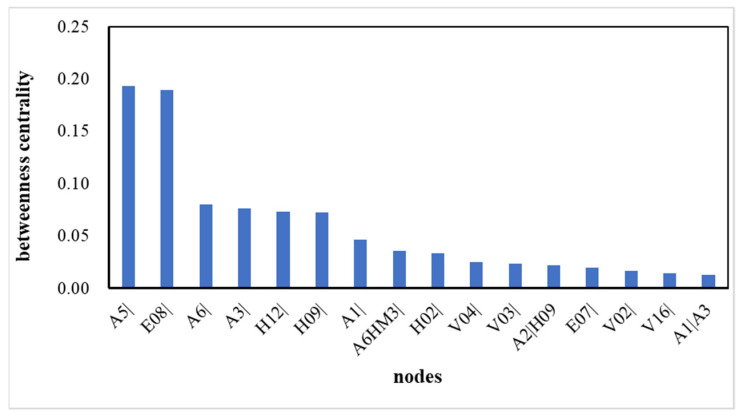
The betweenness centrality of the nodes whose values are greater than 0.01.

Figure 8 gives the betweenness centrality of nodes whose values are greater than 0.01. A5|(Fire) is the most important intermediate causal factor, with a value of 0.1934. To avoid fire accidents, causal factors directedly pointing to A5|(Fire) should be detected, especially those with high frequencies. V12|(Tire overheating), A6HM3|(Leakage and Flammable liquid), and A6|(Leakage) are most likely to cause fire accidents. Meanwhile, A5|(Fire) is most likely to influence A7|(Explosion), A6|(Leakage), and V04|(Tank damaged). If a fire is not detected and controlled, it can lead to explosion, leakage, or tank damage.

E08|(Other road reasons) is the second most important intermediate causal factor, with a value of 0.1892. E08| is likely to point to H09|(Unsafe distance) and A1|(Collision), which illustrates that the road environment can lead to unsafe distances or collisions between vehicles. To avoid accidents caused by E08|, drivers and supercargoes should be familiar with the road environment.

The higher-order nodes of A2|H09 (Scrape|Unsafe distance) and A1|A3 (Collision|Roll-over) are important intermediate causal factors with values of 0.022 and 0.013, respectively. A2|H09 and A1|A3 both likely point to V04|(Tank damaged). This indicates H09→A2→V04 and A3→A1→V04 are two key paths in the HMTACN.

#### 3.2.5. K-Core

K-core is an algorithm for mining subgraphs, in which the number of linkages with other nodes is K. The higher the K value, the more important the subgraph. K-core can contribute to finding condensed subgroups, which plays a key role in the cohesion of the network. In the HMTACN, there were six clusters found by calculating K-core, and Table 2 shows the frequency distribution of cluster values. The frequency of Cluster 2 is the highest, with 109 nodes, which accounts for 44.86%. Cluster 6 contains 18 nodes, and these nodes are closely linked and form a condensed subgroup with a high degree.

**Figure 9:**
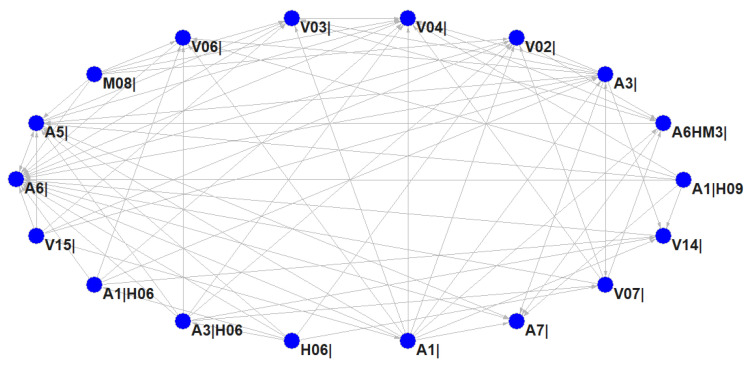
Relationships among 18 nodes in Cluster 6.

Figure 9 presents subgraph of Cluster 6 with detailed relationships among 18 nodes. These 18 nodes consist of 15 first-order nodes and 3 higher-order nodes. The causal factors of the first-order nodes refer to human, vehicle, accident type, and management. For example, improper operation and unsafe driver behaviors are most likely to affect other factors. Unsafe configurations of valves, tanks, and pipes are likely to be influenced by other reasons. Accidents involving collisions, roll-overs, fires, leakages, or explosions are easily triggered and lead to unsafe states. Causal factors of higher-order nodes reveal that collision and roll-over are mainly caused by unsafe distance and improper operation. This indicates that these two unsafe human behaviors are more likely to lead to collision and roll over, which cannot be found by using traditional Markov network models.

## 4. Conclusions

Hazardous material transportation accidents occurring on roads are the results of multiple factors’ complex interactions. In this study, an accident causation model (the HMTACN), based on higher-order networks, was constructed. In the HMTACN, the sequence and dependence of accident causal factors are considered, which are usually neglected in traditional complex networks. The HMTACN is a small-world network, which means that the causal path is short and causal factors closely connect. In general, HMTAs only need four or five steps, and this has not been proposed by previous studies. Therefore, it is better to find initial hazards to control risk communication. Additionally, the HMTACN is a scale-free network, which illustrates that a few causal factors play very significant roles in an HMTA system. Identifying these significant factors and controlling their interactions are quite necessary.

The most significant first-order causal factors mainly focus on accident type, unsafe vehicle state, environment, or unsafe human behavior. Accident type, such as leakage, roll-over, collision, and fire, can be triggered or lead to a domino effect. When these accidents happen, taking immediate action can avoid severe secondary accidents. Meanwhile, unsafe vehicle states, especially those involving tanks, valves, packaging, and pipes, which are directly touching hazardous materials, usually put the hazardous materials in danger. Additionally, when encountering unsafe environments, particularly in rain, snow, or slippery roads, multicar collision prevention, careful driving, and safety warning signs are essential for avoiding accidents. Unsafe human behavior and the most notable causal factors are nonhazmat personnel reasons. This means that not only hazmat transportation workers but also the public should better understand safety. Unsafe behaviors by drivers, especially improper operation, avoidance, and braking, should be corrected, and driver safety training is always necessary. In addition, multiple factors, such as the simultaneous actions of leakage and flammable liquid, which are often overlooked, are equally important and may lead to serious consequences.

Higher-order causal factors can represent the sequence and dependence of interactions among factors, which cannot be found by a traditional Markov network model. Two higher-order causal factors, A2|H09 and A1|A3, are important intermediate casual factors. Meanwhile, higher-order causal factors can represent significant causative paths, which also means that relationships among causal factors are often neglected by existing common accident causation theories. Paths from unsafe distance to scrape, and from roll-over to collision, should be taken seriously as they can lead to more accidents. Furthermore, these two paths mentioned above are most likely to lead to tank damage. When the initial factor experiences an issue, the subsequent factors should immediately receive attention to interrupt risk transmission. In addition, causal factors much more closely connected should receive additional attention as these factors can influence or be influenced by more causal factors.

This study considered the complexity of causation mechanism in an HMTA system. Both significant causal factors and relationships were accurately identified through quantitative analysis. This study proposed an effective way to monitor risks, which is closely geared to the safety management of hazardous materials transportation. This study expanded on accident causation theory and the application of higher-order network models. Finally, although the data used in this paper are current, it is worth pointing out that some important causal factors change with the development of hazardous material transportation systems. Hence, more detailed analysis should be conducted with updated HMTAs data in future research.

## Figures and Tables

**Table 1:** Specific causes of HMTAs.

General Cause	Specific Cause (Code)	General Cause	Specific Cause (Code)
Human (H)	Fatigue driving (H01) Improper avoidance (H02) Fast driving (H03) Improper braking (H04) Improper overtaking (H05) Improper operation (H06) Overload transportation (H07) Distracted driving (H08) Unsafe distance (H09) Unfamiliar with the road (H10) Other hazmat personnel reasons (H11) Non-hazmat personnel reasons (H12)	Vehicle (V)	Tank equipment failure (V01) Valve loose (V02) Valve damaged (V03) Tank damaged (V04) Tank/valve aging (V05) Pipe rupture (V06) Other tank reasons (V07) Blow-out (V08) Transmission shaft fracture (V09) Brake system fault (V10) Vehicle out of control (V11) Tire overheating (V12) Equipment aging (V13) Oil tank damaged (V14) Other vehicle reasons (V15) Packaging issues (V16)
Hazardous Materials (HM)	Explosive materials (HM01) Gas (HM02) Flammable liquid (HM03) Flammable solid (HM04) Oxidizing materials (HM05) Toxic and infectious materials (HM06) Radioactive materials (HM07) Corrosive and irritant materials (HM08) Miscellaneous hazmat (HM09)	Management (M)	Illegally refitting vehicles (M01) No supercargo (M02) Failure to clean tank as required (M03) Illegal transportation (M04) No hazmat qualification certificate (M05) No hazmat transportation license (M06) Inadequate safety check (M07) Other management reasons (M08)
Environment (E)	Downhill road (E01) Slippery road (E02) Turning road (E03) Poor road (E04) Other road reasons (E05) Traffic jam (E06) Multi-car collision (E07) Other traffic reasons (E08) Rain or snow weather (E09) Foggy weather (E10) High temperature (E11) Poor visibility (E12) Other environment reasons (E13)	Accident type (A)	Collision (A1) Scrape (A2) Roll-over (A3) Fall-over (A4) Fire (A5) Leakage (A6) Explosion (A7) Poisoning (A8) Others (A9)

**Table 2:** Frequency distribution of cluster value.

Cluster	Freq	Freq%	CumFreq	CumFreq%
1	58	23.87	58	23.87
2	109	44.86	167	68.72
3	26	10.70	193	79.42
4	18	7.41	211	86.83
5	14	5.76	225	92.59
6	18	7.41	243	100.00

## Data Availability

Not applicable.
